# Proton pump inhibitors reduce the accuracy of faecal immunochemical test for detecting advanced colorectal neoplasia in symptomatic patients

**DOI:** 10.1371/journal.pone.0203359

**Published:** 2018-08-31

**Authors:** Lorena Rodriguez-Alonso, Francisco Rodriguez-Moranta, Claudia Arajol, Pau Gilabert, Katja Serra, Albert Martin, Gemma Ibáñez-Sanz, Victor Moreno, Jordi Guardiola

**Affiliations:** 1 Department of Gastroenterology and Hepatology, University Hospital of Bellvitge-IDIBELL, Barcelona, Spain; 2 Catalan Institute of Oncology, Cancer Prevention and Control Programme, IDIBELL, Barcelona, Spain; University of South Alabama Mitchell Cancer Institute, UNITED STATES

## Abstract

**Background:**

The faecal immunochemical test (FIT) is used in colorectal cancer (CRC) screening and for the detection of advanced colorectal neoplasia (AN) in symptomatic patients, but its accuracy could be improved. Our objective was to assess the impact of proton pump inhibitors (PPI) on the accuracy of the FIT in the detection of AN, namely advanced colorectal adenoma and CRC.

**Methods and findings:**

We performed a prospective study of 1002 individuals referred for a diagnostic colonoscopy at Bellvitge University Hospital from September 2011 through to October 2012. An exhaustive interview was performed by a gastroenterologist, prescription drug dispensing database was reviewed and the patient was given a FIT prior to colonoscopy. The positivity threshold of FIT used was ≥ 20 μg Hb/g feces and the main outcome was AN. AN was detected in 13.2% (133) of patients. The accuracy of FIT for detecting AN in the PPI users and non-PPI users were: sensitivity 43.0% vs 65.6%, P = 0.009; specificity 86.9% vs 92.3%, P = 0.010; and, predictive positive value 34.4% vs 55.5%, P = 0.007, respectively. In multivariate analysis, adjusting for potential confounders, PPIs were associated with false positives in AN detection by FIT (OR 1.63 CI 95% 1.02–2.59, P < 0.037). The ROC curve for the FIT in the detection of AN in the PPI users and non-PPI users was 0.68 (CI 95% 0.61–0.76) and 0.85 (CI 95% 0.79–0.90).

**Conclusions:**

PPI therapy reduces the accuracy of FIT for detecting AN in symptomatic patients.

## Introduction

Periodic faecal occult blood testing, and subsequent colonoscopy if the result is positive, is a widely accepted strategy for colorectal cancer (CRC) screening in average risk population [1–3]. Faecal biochemical tests based on the oxidation of guaiac have been used for this purpose for years resulting in a reduction in CRC-related mortality [4]. Recently, guaiac-based faecal tests are being replaced by faecal immunochemical tests (FIT) that are able to detect smaller amounts of hemoglobin in faeces (60 μg Hb/g faeces vs 10 μg Hb/g faeces) by using antibodies to human globin. FITs are more sensitive for the detection of CRC and advanced neoplasia (AN) in comparison to the guaiac-based faecal tests [1]. Furthermore, recent studies have demonstrated that quantitative FIT is also an objective and accurate method for detecting advanced neoplasia (AN), including advanced adenoma and colorectal cancer, in symptomatic patients. In fact, FIT has shown a better discriminatory ability than lower abdominal symptoms [5–9]. Nevertheless, its accuracy in the detection of advanced adenoma (AA) is far from perfect. The sensitivity of the FIT for CRC is relatively high, at over 85%, but its sensitivity for advanced adenoma (AA) is under 40% [9–11]. AA is associated with a relatively high risk of progression to cancer and is considered the optimal target lesion to prevent colorectal cancer [12–14]. For this reason, improving the diagnostic accuracy of the FIT in the identification of AA and the factors that are likely to influence the accuracy of the FIT is of great importance for the study of symptomatic patients and the CRC screening programme.

Proton pump inhibitors (PPI) are widely prescribed drugs due to their extensive indications, including dyspepsia, gastroesophageal reflux and the prevention of gastrointestinal bleeding in patients on antiplatelet therapy or non steroidal anti-inflammatory drugs (NSAID). PPI are associated with a large number of effects on the gastrointestinal tract [14, 15] that potentially could affect FIT accuracy. Changes in the gastrointestinal microbiome [16], an increase in the incidence of NSAID-induced small bowel injuries [17] and inhibition of pancreatic secretion [18–20] have been described in patients on PPI treatment. Recently, a study performed by Ibañez *et al*. to evaluate the influence of prescription drugs on the accuracy of FIT in colorectal cancer screening found that the use of PPI was associated with false-positive results of FIT [21]. Nevertheless, this study did not include the result of the colonoscopy of patients with a negative FIT and in consequence, the influence of PPI on the sensitivity and specificity of the test could not be ascertained. Therefore, we hypothesized that PPI may modify the precision of the FIT. The objective of this study is to evaluate the impact of PPI treatment on the accuracy of the FIT for detecting AN (AA or CRC).

## Methods

### Study design and patients

This is a post hoc analysis of a previous study that evaluated an urgent referral strategy based on a quantitative FIT for symptomatic patients with suspected colorectal cancer (Rodriguez Alonso *et a*l, 2015) [6]. Briefly, the study included symptomatic patients of more than 18 years of age referred for diagnostic colonoscopy to the Endoscopy Unit of the Bellvitge University Hospital between September 2011 and October 2012, S1 File. Patients referred for adenoma and CRC surveillance, history of previous colectomy, inflammatory bowel disease, polyposis syndromes and hospitalized patients were excluded. Patients with incomplete colonoscopies were only included if its cause was a stenosing neoplasm. Referrals were outpatient requests from general practitioners and community gastroenterologists, as well as hospital requests. An exhaustive questionnaire was administered by a gastroenterologist in a face-to-face interview. In this consultation signs, symptoms, CRC risk factors and, use of medical drugs, including PPI intake and dose were recorded and a specimen collection device of FIT (OC Sensor^®^, Eiken Chemical Co., Ltd., Tokyo, Japan) and the instructions on how to perform and storage the test at home were given [6]. Samples with collection or storage errors were excluded from the study. All tests were analyzed using the OC sensor MICRO desktop analyser (Eiken Chemical ^®^Co., Ltd., Tokyo, Japan). In our study, FIT ≥ 20 μg Hb/g feces was taken as the cut-off value. The endoscopist and the technician were blind to the patient data and FIT results. All colonoscopies were performed by experienced endoscopists. Conscious sedation was administered using intravenous propofol. The colonoscopy was considered complete if caecal intubation was achieved as demonstrated by the visualization of the ileocecal valve or the appendiceal orifice. The bowel preparation was considered adequate according the validated Boston bowel preparation scale. Recorded data included the number, size and histology of polyps and, the presence or absence of CRC. The study protocol was approved by University Hospital of Bellvitge Ethics Committee, reference number PR 283/11 S2 File, and written informed consent was obtained from all patients.

### Outcome measures

The dependent variables analyzed were the detection of AN and CRC. AN was defined as the presence of AA (adenoma ≥ 10 mm, villous component or high-grade dysplasia) or invasive carcinoma. The following independent variables were evaluated for their potential association with AN or CRC: age, gender, tobacco or alcohol use including former or current exposure, family history of CRC, history of colorectal adenoma, dyslipidemia, diabetes mellitus, body mass index, antiplatelet therapy, anticoagulant therapy or NSAID use, iron deficiency anaemia (IDA), abdominal symptoms or signs and, FIT result. Average alcohol consumption (in standard units of alcohol, SUA), was categorized into low-risk and high-risk consumption (> 4 SUA/ day in men and > 2 SUA/day in women) [22]. Abdominal symptoms or signs considered as high risk symptoms, according NICE Guideline criteria were recorded such as patients with IDA (Hb ≤11 g/dl in men or ≤10 g/dl in postmenopausal women), a definitive palpable right-sided abdominal mass or a rectal mass, patients over the age of 40 years with rectal bleeding and diarrhoea for six week, and patients over the age of 60 years with rectal bleeding or diarrhoea for six weeks [23].

### Exposure to proton pump inhibitors

Exposure for each patient was determined from the interview and confirmed with computerized prescriptions recorded. Patients were considered exposed if they intake regularly PPI—omeprazole, lansoprazole, pantoprazole, rabeprazole, or esomeprazole—during the past 90 days or sporadic consume during the past 30 days before the interview date.

### Statistical analysis

Univariate and multivariate analyses were performed to evaluate the accuracy of FIT in the identification of AN and CRC depending on PPI use. The chi-squared test was used to assess the association between categorical data and the detection of AN and CRC in both PPI users and non-PPI users. We evaluated the diagnostic accuracy of positive FIT for advanced neoplasia and colorectal cancer according to proton pumps inhibitors treatment. The outcome measures were sensitivity, specificity, positive predictive value, negative predictive value, overall value, positive likelihood ratio and negative likelihood ratio.

A multivariate analysis based on a forward conditional logistic regression procedure was performed in order to identify independent predictive factors of false positives (FP) of the FIT in the detection of AN and CRC. These factors were included in the multivariable model based on their univariate association with AN and CRC (P < 0.05). Factors not reaching statistical significance were also included if they were considered to be clinically relevant or biologically plausible with a sound scientific rationale. The results of the model are reported as adjusted odds ratios (OR) and their 95% confidence intervals (CI). Statistical analysis was carried out using SPSS, Version 17, Inc, Chicago, IL.

## Results

### Descriptive findings

During the study period, 1003 patients were enrolled in the study. One individual was excluded due to the pharmacological history of the patient could not be confirmed in the electronic prescription registry. Finally, the data of 1002 patients were analyzed. As described in detail elsewhere [6], 133 patients were found to have AN (13.3%), including 103 patients with advanced adenoma (10.3%) and 30 patients with CRC (3.0%). Non-advanced adenoma was identified in 168 patients (16.8%). Colonoscopy resulted normal in 600 patients (59.8%) and other conditions, such as inflammatory or vascular lesions, were found in 101 patients (10.1%). A total of 398 (39.7%) patients were chronic PPI users, 157 (15.7%) due to prevention of gastrointestinal damage in patients on antiplatelet or NSAID therapy and 241 (24.0%) due to different conditions such as dyspepsia or gastroesophageal reflux disease. One hundred and twenty-seven (12.7%) patients were sporadic users due to episodic treatment of gastroesophageal reflux disease. A total of 525 (52.4%) patients were considered as PPI users. Demographic and clinical characteristics of patients and endoscopic findings according to PPI use are shown in Table 1. There were significant differences in age, smoking status, dyslipidemia, diabetes and the therapy with NSAIDs or antiplatelet agents between the PPI and the non-PPI users.

**Table 1:** Demographic and clinical characteristics and endoscopic findings of patients according to PPI use.

Variable	PPI users	Non PPI users	*P* Value
**PPI therapy, n (%)**	525 (52.4)	477 (47.6)	
**Age (years), mean +/- SE**	64.9 ± 11.3	57.3 ± 14.0	< 0.001
**Male sex, n (%)**	232 (44.2)	238 (49.8)	0.071
**Smoking status (former or current), n (%)**	228 (43.4)	235 (49.2)	0.047
**High risk consumption of alcohol, n (%)**	108 (20.6)	108 (22.6)	0.327
**Dyslipidemia, n (%)**	283 (53.9)	168 (35.2)	< 0.001
**Diabetes Mellitus, n (%)**	137 (26.1)	60 (12.5)	< 0.001
**Body mass index ≥ 30kg/m^2^, n (%)**	147 (28.0)	107 (22.4)	0.100
**NSAID/antiplatelet agents users, n (%)**	182 (34.6)	63 (13.2)	<0.001
**FIT ≥ 20 μg Hb/g, n (%)**	90 (17.1)	70 (14.6)	0.379
**Size of Advanced Adenoma (mm), mean +/- SE**	12.1± 8.4	13.0± 7.8	0.640
Proximal location of Advanced Neoplasia*, n (%)	30 (5.7)	18 (3.8)	0.203
**Advanced Neoplasia, n (%)**	72 (13.7)	61 (12.8)	0.666
**Advanced adenoma, n (%)**	57 (10.8)	46 (9.6)	0.528
**Colorectal cancer, n (%)**	15 (2.8)	15 (3.1)	0.790

NSAID: non steroidal anti-inflammatory drugs,

*Right colon lesions

The prevalence of high risk symptoms according NICE Guideline criteria by PPI use are shown in Table 2. There were significant differences between the PPI and the non-PPI users in the prevalence of IDA (18.6 vs 5.4, *P* < 0.001) and in the prevalence of patients over the age of 60 years with rectal bleeding for six weeks without anal symptoms (6.1 vs 3.1, *P* = 0.027).

**Table 2:** Prevalence of high risk symptoms according NICE Guideline criteria by PPI use.

Nice Guideline Criteria	PPI users n (%)	Non PPI users n (%)	*P* Value
	525 (52.4)	477 (47.6)	
**Patients over the age of 40 years with rectal bleeding and diarrhoea for six week**	23 (4.3)	26 (5.4)	0.433
**Patients over the age of 60 years with rectal bleeding for six weeks without anal symptoms**	32 (6.1)	15 (3.1)	0.027
**Patients over the age of 60 years with diarrhoea for six weeks**	66 (12.6)	47 (9.8)	0.174
**Definitive palpable right-sided abdominal mass**	1 (0.01)	0 (0)	0.340
**Rectal mass**	4 (0.07)	1 (0.02)	0.215
**Iron deficiency anemia**	94 (18.6)	26 (5.4)	< 0.001

### Diagnostic accuracy of FIT according to PPI treatment

The diagnostic accuracy of the FIT for the detection of AN in the whole population was: sensitivity 53.4%; specificity 89.4%; predictive positive value (PPV) 43.6%, negative predictive value (NPV) 92.6%, positive likelihood ratio (PLR) 5.0 and negative likelihood ratio (NLR) 0.5. The diagnostic accuracy of the FIT in the PPI users and non- PPI user is showed in Table 3. The PLR and NLR in PPI users were: 3.3 and 0.6, respectively. The PLR and NLR in non—PPI users were: 8.5 and 0.4, respectively.

**Table 3:** Diagnostic accuracy of positive faecal immunochemical test (FIT ≥ 20 μg/g) for advanced neoplasia according to PPI treatment.

	PPI users	Non PPI users	*P*=
Sens	43.0	65.6	0.009
Spec	86.9	92.3	0.010
PPV	34.4	55.5	0.007
NPV	90.5	94.8	0.019
OV	80.9	88.8	

PPI: proton pump inhibitor, Sens: sensitivity, Spec: specificity, PPV: positive predictive value, NPV: negative predictive value, OV: overall value.

The diagnostic accuracy of the FIT for the detection of CRC in the whole population was: sensitivity 93.3%; specificity 86.1%; PPV 17.2%, NPV 99.8%, PLR 6.7 and NLR 0.5. The diagnostic accuracy of the FIT in the PPI users and non- PPI user is showed in Table 4. The PLR and NLR in PPI users were: 6.3 and 0.1, respectively. The PLR and NLR in non—PPI users were: 7.4 and 0.1, respectively.

**Table 4:** Diagnostic accuracy of positive faecal immunochemical test (FIT ≥ 20 μg/g) for colorectal cancer according to PPI treatment.

	PPI users	Non PPI users	*P* =
Sens	93.3	93.3	0.759
Spec	85.1	87.4	0.289
PPV	15.5	19.4	0.515
NPV	99.7	99.7	0.760
OV	85.3	87.6	

PPI: proton pump inhibitor, Sens: sensitivity, Spec: specificity, PPV: positive predictive value, NPV: negative predictive value and OV: overall value.

The diagnostic accuracy of the FIT for advanced adenoma is provided in the Supplementary Table 1. The area under the ROC curves for the FIT in the detection of AN, AA and CRC was 0.76 (CI 95% 0.71–0.81), 0.68 (CI 95% 0.63–0.75) and 0.94 (CI 95% 0.91–0.96), respectively. The ROC curve for the FIT in the detection of AN in the PPI users and non-PPI users was 0.68 (CI 95% 0.61–0.76) and 0.85 (CI 95% 0.79–0.90), respetively. The ROC curve for the FIT in the detection of AA in the PPI users and non-PPI users was 0.60 (CI 95% 0.51–0.68) and 0.79 (CI 95% 0.72–0.86), respectively. The ROC curves for the FIT in the detection of CRC in the PPI—users and non-PPI users were 0.94 (CI 95% 0.92–0.97) and 0.93 (CI 95% 0.89–0.97), respectively.

#### Evaluation of false positives

In our study, the false positive (FP) rate produced by the FIT for the detection of AN and CRC were 10.6% and 13.9%, respectively. In univariate analysis, the proportion of FIT FP results for the detection of AN was higher in the PPI users than in the non-PPI users (13.1% vs 7.7%; *P* = 0.013). The presence of rectal bleeding and diarrhea over 40 years old patients (18.3% vs 8.6%; P = 0.020) and the presence of IDA (15.8% vs 8.9%; P = 0.006) were also associated with FIT FP results for the detection of AN. Other high risk signs or symptoms according NICE criteria as well as gender, dyslipidemia, smoking status, high risk consumption of alcohol, diabetes, NSAID/antiplatelet agents users were not associated with FIT FP results for the detection of AN.

In the multivariate analysis for the diagnosis of AN, PPIs therapy, IDA and rectal bleeding and diarrhea over 40 years old patients were associated with a FIT FP result after adjusting for age, gender, smoking status, dyslipidemia, diabetes, NSAIDs and antiplatelet therapy and other high risk symptoms according NICE criteria (see Table 5). Furthermore, in patients without AN (n = 869), the faecal haemoglobin (f-Hb) concentration mean was higher in the PPI-users (n = 453) than in the non-PPI users patients (n = 416) (median and interquartile ranges were: 1.0 μg Hb/g feces [0–5.4] vs 0.2 μg Hb/g feces [0–4.0]; *P* = 0.005).

**Table 5:** Multivariate predictors of false positive result of faecal immunochemical test (FIT ≥ 20 μg/g) for diagnosing advanced neoplasia.

Variable	OR (95% CI)	*P* Value
**Iron deficiency anaemia**	1.84 (1.05–3.23)	0.032
**Rectal bleeding and diarrhea over 40 years old**	*2*.*46 (1*.*15–5*.*32)*	*0*.*021*
**PPI treatment**	*1*.*63 (1*.*02–2*.*59)*	*0*.*037*

PPI: proton pump inhibitors

Regarding the detection of colorectal cancer, male gender and the presence of IDA were both associated with FIT FP results (15.5% vs 11.4%; *P* = 0.037 and 19.1% vs 12.6%; *P* = 0.038, respectively). The proportion of FITFP results for the detection of colorectal cancer was also higher in PPI users than in non-PPI users, but the difference was not statistically significant (14.9% vs 12.5%; *P* = 0.282).

#### Evaluation of false negatives

In our study, the false negative (FN) rate produced by FIT for the detection of AN and CRC were 46.6% and 6.6%, respectively. The FIT FN rate for the detection of AN was higher in the PPI users than in the non-PPI users (56.3% vs 35.5%; *P* = 0.024). In the multivariate analysis, PPI treatment was not associated with a FIT FN result after adjusting for age, gender, IDA and, NSAIDs and antiplatelet therapy. Nevertheless, in patients with AN (n = 133), the f-Hb concentration was lower in the PPI-users (n = 70) than in the non-PPI users patients (n = 60) (median and interquartile ranges were: 15.4 μg Hb/g feces [0.1–132.7] vs 64.2 μg Hb/g feces [3.5–192.1], *P* = 0.047). In addition, in patients with advanced adenoma, excluding CRC (n = 103), f-Hb concentration was lower in the PPI-users (n = 56) than in the non-PPI users patients (n = 45) (median and interquartile ranges were: 4.2 μg Hb/g feces [0.0–35.2] vs 26.1 μg Hb/g feces [2.1–139.7], *P* = 0.025).

## Discussion

Identifying the factors that modify the accuracy of FIT could improve its usefulness as a biomarker of significant colorectal disease. Our study demonstrates that the accuracy of FIT in the detection of AN clearly decreases in symptomatic patients undergoing PPI treatment.

Recently, a systematic review was performed by Westwood *et al* to evaluate the diagnostic performance of FIT to detect AN and CRC in patients with lower abdominal symptoms. In this study the sensitivity and the specificity summary estimate of FIT ≥ 20 μHb/g for AN were 64% and 86%, respectively, consistent with the sensitivity and the specificity values found in our study, 53% and 89%, respectively. Regarding CRC, the sensitivity and the specificity summary estimate of FIT ≥ 20 μHb/g were 93% and 86%, respectively, closely similar to our results of 93% sensitivity and 87% specificity [24]. Similarly, the accuracy of FIT ≥ 20 μHb/g for CRC found in our study was also comparable to that found in asymptomatic average risk population in CRC screening programs (89% sensitivity and 91% specificity) [25].

PPIs are the first line treatment for many gastrointestinal conditions and this therapy is often overprescribed [26]. In line with this, more than half of the patients (52.4%) in our series were on PPI treatment. Recently, a variety of effects of PPIs on the small and large bowel have been described. Its use has been found to predispose patients to NSAID induced small bowel injury, promote changes in the gut microbiome and inhibit pancreatic secretion [15–20]. To date, there are no studies that evaluate the relationship between PPI treatment and the accuracy of FIT in the diagnosis of AN. We have found that the proportion of FP and FN FIT results in the detection of AN were significantly higher in the PPI users than in the non-PPI users (11.2 vs 6.7; *P* = .013 and 7.7 vs 4.3; *P* = 0.024, respectively). In patients with AN the mean value of f-Hb is significantly lower in the PPI users than the non-PPI users (15.4 vs 64.2; *P* = 0.047) that leads to FN results. Therefore, in patients with AN who are undergoing treatment with PPI, the median value of f-Hb is lower than the value taken as the cut off, resulting in an increased number of FN results.

Several factors have been associated with FP results in the FIT in screening programme for CRC, including female gender and younger age [27]. A recently published study by Ibáñez-Sanz *et al*, identified female gender, successive screening, haemorrhoids or anal fissure, and PPI use to be risk factors for FP results in CRC screening programme [21]. Their results are consistent with those of our study. We found that PPI use was an independent variable associated with FP of FIT in the detection of AN after adjusting for age, gender, smoking status, dyslipidemia, diabetes and NSAIDs and antiplatelet agents use. The mechanism by which PPI therapy impairs the accuracy of FIT in the detection of AN is unknown but several possibilities may be hypothesized. Firstly, PPI treatment could be a surrogate of small-bowel NSAID induced injury. Several studies, including an RCT, reported a raised incidence of NSAID induced injuries in PPI users [28]. Small intestinal dysbiosis caused by the marked suppression of gastric acid secretion has been implicated in this effect. In addition, bacterial genes associated with epithelial invasion have been identified after PPI treatment in healthy subjects [29–31]. This effect could play a role in the loss of accuracy of FIT. Secondly, PPI treatment could impair the organ specificity of the FIT. The FIT has a high degree of selectivity for colorectal bleeding due to the fact that human globin from lesions located proximal to the colon are readily degraded by proteases and do not positivize the test. Wang *et al* demonstrated that PPI use inhibits pancreatic secretion in both rat and human pancreatic cells [20]. It might be argued that the FIT, in patients undergoing PPI treatment, is able to detect undigested globin from upper GI bleeding, resulting in a false positive.

Conversely, the increase of false negative FIT results in patients with PPI and AN was a unexpected finding without consistent explanation and for which a fortuitous association cannot be excluded. Therefore, this data should be taken with caution and should be confirmed in subsequent studies.

The strengths of this study include its prospective design, which provides endoscopic information about both FIT positive and FIT negative patients. Despite the availability of studies dealing with the accuracy of the FIT, many are performed in screening programs and endoscopic information is only available from FIT positive patients. Factors affecting FN are difficult to study given that it requires performing colonoscopies in patients with negative FIT. Another of the strengths of the study was that the use of drugs was rigorously evaluated through the initial interview and confirmed in the electronic prescription registry.

This study has several limitations. Firstly, it was performed in a tertiary care hospital, which may lead to a selection bias [6]. Nevertheless, most of the patients were referred from primary care and the rate of ANs found (13.3%) was similar to that found in other open-access endoscopy units in our region. Secondly, the fact that the study population only included symptomatic patients already referred for colonoscopy may be another source of bias. However, ours is an open-access endoscopy unit that belongs to the public health service and referral physicians have a low threshold for the referral of patients for colonoscopy. Third limitation is the small number of CRC. We found a significant difference in accuracy of FIT between PPI users and no-PPI users for the detection of advanced adenoma and advanced neoplasia but not for detecting CRC, presumably because of the small number of CRC cases. Finally, our results might not be generalizable to CRC screening population. The characteristics of the neoplastic lesions found in the asymptomatic average risk population may differ from those found in our symptomatic population leading to differences in the accuracy of the FIT due to a spectrum effect [32].

In summary, our study shows that PPI therapy impairs the performance of FIT for the detection of AN in symptomatic patients. Given the widespread use of these drugs in the general population, the negative impact on screening CRC programs may be substantial. The effect of PPI therapy need to be investigated in screening population due to this therapy could modify the accuracy of FIT in a CRC screening programme. Further studies are required to confirm these findings and to determine whether cut-off values of FIT should be modified in PPI users or whether PPI should be discontinued (and when) before FIT testing.

## Supporting information

S1 Table Diagnostic accuracy of positive faecal immunochemical test (FIT ≥ 20 μg/g) for advanced adenoma according to proton pumps inhibitors treatment. (DOCX)

S1 File Univerisity Hospital of Bellvitge Ethics Committee. (PDF)

S2 File Anonymized data set of the study. (SAV)
