# Supplementary material for: Proton pump inhibitors reduce the accuracy of faecal immunochemical test for detecting advanced colorectal neoplasia in symptomatic patients
Source: PLoS One. 2018 Aug 31;13(8):e0203359. doi: 10.1371/journal.pone.0203359 (PMC6118361; doi:10.1371/journal.pone.0203359)
Supplement: S1 Table — (DOCX) [file pone.0203359.s001.docx]

S1 table. Diagnostic accuracy of positive faecal immunochemical test (FIT ≥ 20 μg/g) for advanced adenoma according to proton pumps inhibitors treatment.

| Variable | **Sens** | **Spec** | **PPV** | **NPV** | **OV** | **PLR** |
| --- | --- | --- | --- | --- | --- | --- |
| Overall patients | 41.7 | 86.8 | 26.5 | 92.9 | 82.1 | 3.5 |
| PPI users | 29.8 | 84.4 | 18.9 | 90.8 | 78.4 | 1.9 |
| Non - PPI users | 56.5 | 89.3 | 36.1 | 95.1 | 86.2 | 5.3 |

FIT: Faecal immunochemical test, PPI: proton pump inhibitor, Sens: sensitivity, Spec: specificity, PPV: positive predictive value, NPV: negative predictive value, OV: overall value, PLR: positive likehood ratio, NLR: negative likehood ratio.
