# Supplementary material for: Proton pump inhibitors reduce the accuracy of faecal immunochemical test for detecting advanced colorectal neoplasia in symptomatic patients
Source: PLoS One. 2018 Aug 31;13(8):e0203359. doi: 10.1371/journal.pone.0203359 (PMC6118361; doi:10.1371/journal.pone.0203359)
Supplement: S1 File — (PDF) [file pone.0203359.s002.pdf]

INFORME DEL COMITÉ ÉTICO DE INVESTIGACIÓN CLÍNICA SOBRE PROYECTOS DE INVESTIGACIÓN

El Comité Ético de Investigación Clínica del Hospital Universitari de Bellvitge, en su reunión de fecha 1 de Diciembre de 2011 (Acta 21/11), tras examinar toda la documentación presentada sobre el proyecto de investigación con nuestra ref. **PR283/11**, titulado:

"ADECUACIÓN DEL USO DE LA COLONOSCOPIA PARA EL DIAGNÓSTICO DE CÁNCER COLORRECTAL BASADO EN INMUNOTEST"

Presentado por el Dr. Francisco Rodríguez Moranta del Servicio del Aparato Digestivo (Unidad de Enfermedad Inflamatoria Intestinal) del Hospital Universitari de Bellvitge – Fundació IDIBELL, como investigador principal, ha acordado emitir INFORME FAVORABLE al mencionado proyecto.

Bellvitge Hospital
Comitè Ètic d'Investigació Clínica

Fdo. Dr. Enric Sospedra Martínez

Secretario del CEIC

L'Hospitalet de Llobregat, 1 de Diciembre de 2011
